# Contextual challenges in implementing artificial intelligence for healthcare in low-resource environments: insights from the SPEC-AI Nigeria trial

**DOI:** 10.3389/fcvm.2025.1516088

**Published:** 2025-03-11

**Authors:** Demilade A. Adedinsewo, Damilola Onietan, Andrea Carolina Morales-Lara, Serin Moideen Sheriff, Bosede B. Afolabi, Oyewole A. Kushimo, Amam C. Mbakwem, Kehinde F. Ibiyemi, James Ayodele Ogunmodede, Hadijat Olaide Raji, Sadiq H. Ringim, Abdullahi A. Habib, Sabiu M. Hamza, Okechukwu S. Ogah, Gbolahan Obajimi, Olugbenga Oluseun Saanu, Solomon Aborisade, Olusoji E. Jagun, Francisca O. Inofomoh, Temitope Adeolu, Kamilu M. Karaye, Sule A. Gaya, Yahya Sa’ad, Isiaka Alfa, Cynthia Yohanna, Peter A. Noseworthy, Rickey E. Carter

**Affiliations:** ^1^ Department of Cardiovascular Medicine, Mayo Clinic, Jacksonville, FL, United States; ^2^ Department of Obstetrics and Gynaecology, College of Medicine and Centre for Clinical Trials, Research and Implementation Science, University of Lagos, Lagos, Nigeria; ^3^ Cardiology Unit, Department of Medicine, Lagos University Teaching Hospital, Lagos, Nigeria; ^4^ Department of Obstetrics & Gynaecology, University of Ilorin Teaching Hospital, Ilorin, Kwara, Nigeria; ^5^ Department of Medicine, University of Ilorin, Ilorin, Nigeria; ^6^ Department of Medicine, Rasheed Shekoni Specialist Hospital, Dutse, Jigawa, Nigeria; ^7^ Department of Obstetrics and Gynaecology, Rasheed Shekoni Teaching Hospital, Dutse, Jigawa, Nigeria; ^8^ Department of Medicine, University of Ibadan, Oyo, Nigeria; ^9^ Department of Obstetrics and Gynaecology, University College Hospital Ibadan, Oyo, Nigeria; ^10^ Department of Obstetrics and Gynaecology, Olabisi Onabanjo University Teaching Hospital, Sagamu, Ogun, Nigeria; ^11^ Cardiology Unit, Department of Medicine, Olabisi Onabanjo University Teaching Hospital, Sagamu, Ogun, Nigeria; ^12^ Department of Medicine, Bayero University and Aminu Kano Teaching Hospital, Kano, Nigeria; ^13^ Department of Obstetrics and Gynaecology, Bayero University/Aminu Kano Teaching Hospital, Kano, Nigeria; ^14^ Lakeside Healthcare at Yaxley, the Health Centre, Peterborough, United Kingdom; ^15^ Department of Cardiovascular Medicine, Mayo Clinic, Rochester, MN, United States; ^16^ Department of Quantitative Health Sciences, Mayo Clinic, Jacksonville, FL, United States

**Keywords:** artificial intelligence, cardiomyopathy, electrocardiogram, implementation science, resource-limited settings, pregnancy, Nigeria

## Abstract

Nigeria is the most populous country in Africa with the highest gross domestic product (GDP) as of 2022. However, Nigeria is burdened by significant health challenges including an extremely high maternal mortality ratio, inadequate human resources, poor healthcare infrastructure, and population-level poverty rates as high as 40%. Nigeria also has the highest reported prevalence of peripartum cardiomyopathy worldwide which contributes to maternal mortality. Unfortunately, the diagnosis of peripartum cardiomyopathy is often delayed and mortality rates following diagnosis are extremely high (approximately 50%). Thus, there is a huge unmet need for simple, effective, and accessible solutions for cardiomyopathy detection in this population. To address maternal mortality through screening and early diagnosis, we designed and conducted a randomized controlled clinical trial (NCT05438576) of an artificial intelligence (AI) technology in Nigeria. The objective of the study was to evaluate the impact of AI-guided screening on cardiomyopathy detection in obstetric patients. The study findings showed AI-guided screening doubled the detection of cardiomyopathy (defined as left ventricular ejection fraction <50%) when compared to usual care with a number needed to screen of 47. As we explore next steps in relation to deploying this technology for clinical use in Nigeria, we sought to gather contextual information and broadly share lessons learned from the recently completed trial. To that end, we convened a round table discussion with all study site investigators aimed at identifying site-specific contextual challenges related to the development and conduct of the study. The SPEC-AI Nigeria study is the first published randomized controlled clinical trial of a health AI intervention in Nigeria. Insights gained from this study can inform future AI intervention studies in clinical care, guide the development of implementation strategies to ensure effective interventions are successfully incorporated into clinical care, and provide a roadmap for key stakeholders to consider when evaluating AI-technologies for use in low-resource settings.

## 1 Current healthcare landscape and the use of artificial intelligence for clinical care in Nigeria

Nigeria is a lower-middle-income country with the largest population in Africa (1) (approximately 224 million persons as of 2023) (2). It had the largest economy with the highest gross domestic product (GDP) in Africa as of 2022 (3) and has been identified as an emerging world power (4). Nigeria continues to face significant health-related challenges resulting in poor health indices. Notably it had the highest absolute number of maternal deaths globally totalling 82,000 in 2020 (5). Peripartum cardiomyopathy, a unique form of pregnancy related heart failure is also prevalent, estimated at approximately 1 in 96 live births in Northern Nigeria (6) (the highest incidence reported worldwide) which also contributes to adverse maternal health outcomes.

Nigeria has been described as having a double burden of disease characterized by high rates of both infectious and non-communicable diseases (7) which puts significant strain on a dysfunctional healthcare system. As such, a multi-sectoral approach is needed to improve healthcare delivery and outcomes.

Advancements in digital technologies, such as artificial intelligence (AI) have the potential to leapfrog Nigeria's economy and the health sector into 21st century (8). Government efforts to drive AI innovation include the establishment of the National Center for Artificial Intelligence and Robotics (NCAIR). Additionally, the Federal Ministry of Communications, Innovation, and Digital Economy (FMCIDE) has begun drafting a national AI strategy (9) to leverage AI as a driver for sustainable development. There are several AI start-ups in Nigeria primarily focused on financial technology, agriculture, digital identity and chatbot development, with fewer in the healthcare sector (10). FMCIDE has also launched a national broadband plan aimed at improving access to healthcare in rural areas through telehealth platforms (11). However, evidence from rigorous validation studies and prospective clinical trials assessing the effectiveness and impact of AI-based solutions on health outcomes remains limited.

## 2 Contextual barriers to healthcare AI use in Nigeria and suggested strategies for implementation

The Screening for Peripartum Cardiomyopathies Using Artificial Intelligence (SPEC-AI) Nigeria trial (12) (NCT05438576) was designed to evaluate the impact of AI guided screening for pregnancy-related cardiomyopathies on clinical outcomes in the peripartum period.

**Figure 1:**
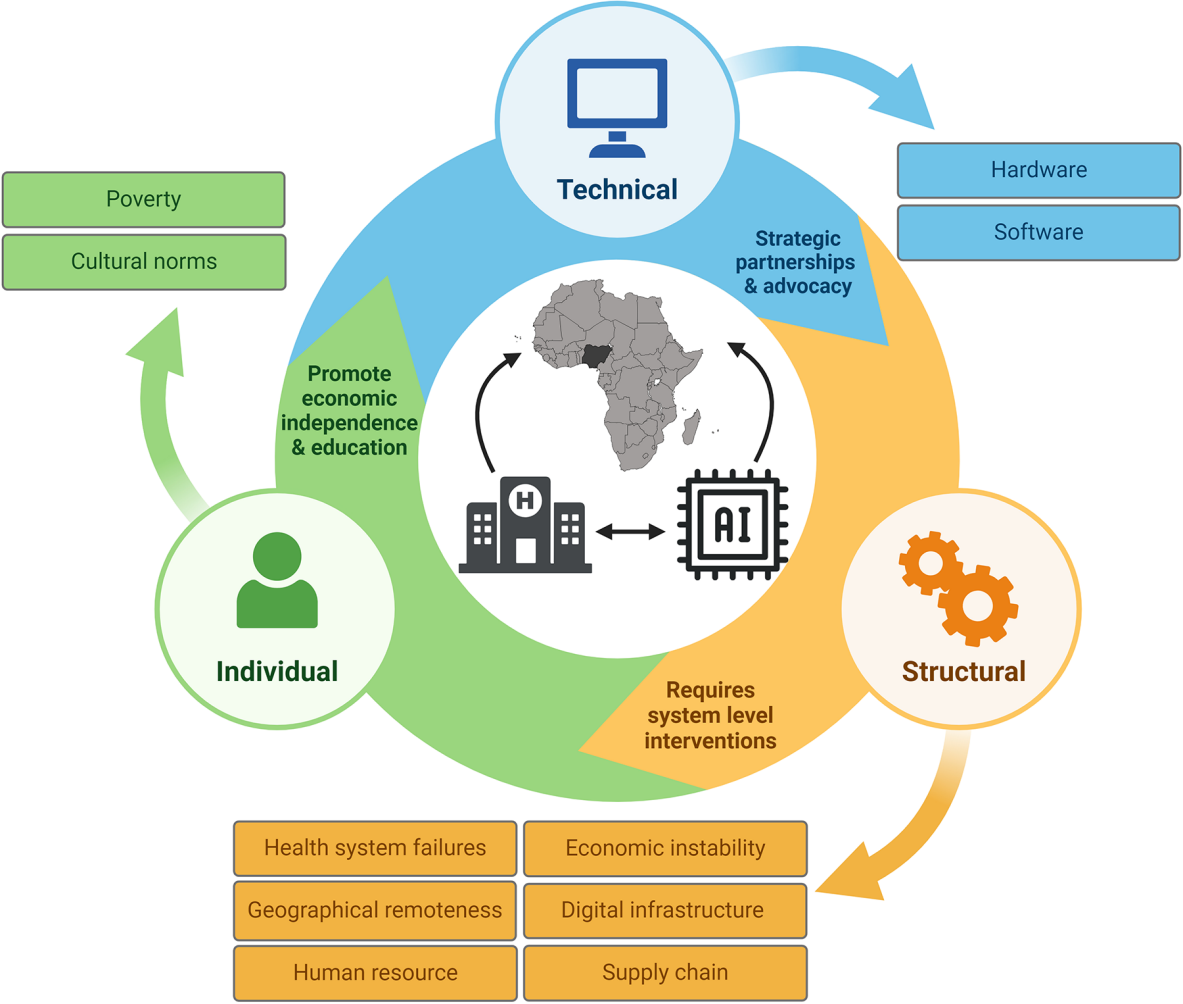
Barriers to implementing artificial intelligence for healthcare in resource-limited settings. This figure broadly identifies structural, technical, and individual barriers to AI implementation based on lessons learned from the SPEC-AI (Screening for Peripartum Cardiomyopathies using Artificial Intelligence) Nigeria study.

In this article, we explore structural, individual, and technical factors that impact the implementation of AI in clinical care in Nigeria (Figure 1). Drawing primarily on our experience in developing and conducting the SPEC-AI Nigeria study (13), we propose solutions and strategies to address these barriers. The six hospitals involved in this study were: Aminu Kano Teaching Hospital (AKTH), Lagos University Teaching Hospital (LUTH), Olabisi Onabanjo University Teaching Hospital (OOUTH), Rasheed Shekoni Specialist Hospital (RSSH), University College Hospital Ibadan (UCH) and University of Ilorin Teaching Hospital (UITH). All sites were teaching hospitals and tertiary centers, with a combined total of 27 cardiologists and 77 obstetricians on staff.

### 2.1 Structural/societal factors

A.
*Health system failures:*

Multiple challenges related to the health system and associated infrastructure in Nigeria were identified.

(1) Limited availability of essential diagnostic imaging equipment or the use of outdated machines significantly hinders healthcare professionals in providing the highest quality of care to patients. Two of the sites with standard echocardiographic machines (equipped with advanced ultrasound capabilities such as 2-dimensional volumetric analysis and or speckle tracking) acquired these through philanthropic donations or research grants.
(2) Lack of stable electric power supply and fluctuations in voltages significantly impact equipment functionality. To address this, many sites had purchased voltage stabilizers to mitigate these effects. These stabilizer systems often include uninterruptible power supply (UPS) functions, providing backup power for a few minutes during grid failures, which are unfortunately common in Nigeria. Frequent power supply interruptions often led to echocardiograms needing to be re-scheduled. This can be frustrating for both healthcare teams and study participants, contributing to attrition during the study. Although many sites had alternative power sources such as a gasoline or diesel generator, there were concerns about using these to power medical devices because voltage fluctuations from the generator contributes to equipment damage and the need for frequent repairs.
(3) Limited use of electronic medical records made data abstraction and identification of adverse clinical outcomes from clinical documents challenging. Five out of six sites continue to utilize paper-based medical records with 4 sites relying exclusively on them. LUTH began the transition to electronic medical records in 2023; however, it was not fully integrated into clinical practice during the study period. OOUTH has completely migrated to electronic medical record documentation; however, existing paper records have not been integrated with the electronic system, resulting in clinicians frequently consulting paper records for continuity of care. Inefficient hospital processes due to non-digitization also poses unique challenges. For example, locating a patient's medical record files can take several minutes and patients often spend excessive time waiting in line for clinical tests. Additionally, all sites required various fragmented administrative procedures and paperwork as part of the care process.

#### Proposed solution to health system failures

Suggested strategies for interventions at the health system level include (1) the use of portable battery-powered solutions to address the need for diagnostic imaging. A unique strength of the AI-guided screening intervention was the portable, battery-powered digital stethoscope. This device can be transported easily across clinical locations, does not rely on electric power supply and provides real-time results, allowing decisions to be made at the point of care. To address echocardiographic needs, the use of portable devices to obtain a focused cardiac ultrasound (FOCUS) represents a potential solution. While portable ultrasound technology has been available for many years, its routine clinical use has been hampered by the inability to acquire sufficient diagnostic images without extensive training. Novel solutions to address this challenge now exist with the use of AI-guidance to support ultrasound novices in acquiring diagnostic FOCUS images (14, 15). One potential strategy is to deploy a dual intervention: portable AI-powered digital stethoscope for population-based initial screening and risk stratification, paired with a portable ultrasound device with AI-guidance to support echo image acquisition and estimation of ejection fraction (16) for those who screen positive to facilitate the timely initiation of medical therapy (2). In the long term, investing in alternative renewable energy sources such as solar power (17) will be essential. This investment will ensure sustainability of a digitized healthcare system (3). The transition to electronic medical records has the potential to improve care processes as well as longitudinal patient care. Potential solutions to convert existing handwritten paper records into digitized formats include the use of novel optical character recognition technology in addition to natural language processing tools (18).

*B. Geographical remoteness:*

Was another major challenge. For patients receiving obstetric care, long distances to the healthcare facility causes a considerable strain on their overall wellbeing and negatively impacted their ability to attend all recommended clinical care visits. This concern was particularly pronounced for women receiving care at RSSH and UITH.

#### Proposed solution to geographical remoteness

Adopting hybrid forms of care can address this challenge. These may include in-person visits in addition to telemedicine visits or alternatively home visits (by nurses or community health workers) in low-risk patients. An obstetric care model involving a reduced number of in-person visits has been evaluated in the United States and shown to be acceptable and effective with higher patient satisfaction compared to traditional in-person prenatal visits (19, 20).

*C. Human resource shortages:*

Many healthcare institutions in Nigeria do not have a licensed cardiologist (21). For centers who do, there are a limited number of trained specialists (cardiologists and sonographers). Only one of six sites (LUTH) utilized sonographers for echocardiographic image acquisition. The lack of sonographers means cardiologists and resident physicians need to make time to acquire and interpret echocardiographic images in addition to their busy workload. This results in care inefficiencies and further limits access to a cardiovascular specialist as clinic schedules are closed on certain days due to the need for imaging. All sites were high-volume centers with busy outpatient cardiology and obstetric care clinics with each physician seeing an average of 20–40 patients daily in clinic alone. Recruiting and retaining clinical trainees (cardiology and obstetrics) is also a challenge. Factors contributing to this include poor remuneration, insufficient career development support, unsatisfactory working conditions, and limited future employment prospects in academic medical centers. Highly qualified trainees often emigrate to other countries (22), contributing to the health workforce brain drain (3). The doctor-to-patient ratio in Nigeria is one of the lowest in sub-Saharan Africa, estimated as 10 doctors per 100,000 population compared to the regional average of 17 doctors per 100,000 as of 2010 (3). According to a 2019 estimate from the Medical and Dental Council of Nigeria (MDCN), the doctor-to-patient ratio in rural areas is as low as 1 in 22,000 (23). This estimate has almost certainly worsened due to a significant loss of skilled healthcare workers in Nigeria over the past few years (24).

#### Proposed solution to human resource shortages

A multi-faceted approach is required to recruit and retain skilled health care workers. Interim solutions include the use of technology solutions and task shifting in low-risk patients to improve care efficiency. A successful example of task shifting has been demonstrated in studies based in Ghana which utilized trained nurses for blood pressure management supported by remote blood pressure monitoring (25). Another example incorporated nurse led virtual obstetric care visits (phone or online) to reduce the number of in-person visits for prenatal care (19). The use of AI technology for echocardiographic image acquisition (14, 15) can temporarily address the need for trained sonographers in low-resource settings, and images can be interpreted remotely by a cardiologist in a different location. There are existing clinical care models that allow trained physicians to provide remote reading and interpretation services for ECGs, echocardiograms, and radiological images.

*D. Economic instability*

During the study (August 2022 – May 2024), Nigeria plunged into an economic crisis, beginning in 2023. This was partly driven by federal government reforms that led to an astronomical rise in inflation rates and depreciating currency values with consequent marked increase in the cost of imported goods (26), a decline in economic growth, and millions of citizens being pushed into poverty (27). Poor economic conditions combined with catastrophic healthcare expenses due to a health system that largely relies on out-of-pocket payments (3), worsened access to care and patient engagement with health services.

#### Proposed solution to economic instability

Ideally, the optimal solution involves creating and adopting sustainable economic reforms, overhauling of the health system infrastructure, and strengthening of health-related federal agencies. Achieving these goals requires strong political will and responsible governance, reflecting a “whole-of-government approach” (3). Interim strategies include flexible and cost-effective care solutions such as hybrid care options (19), task-shifting (25), and technology-based interventions (13) as previously described.

*E. Digital infrastructure, Literacy, and Internet connectivity*:

Internet services are significantly limited in many parts of Nigeria with mobile internet being the main form of connectivity. Although internet adoption in Nigeria has increased over the years, basic broadband (low-speed internet) penetration rate was only 31% as of 2018 (11). The cost and the poor quality of internet services contributes to low uptake (28). Study sites invested in purchasing portable Wi-Fi hotspot devices with subscriptions from a local mobile internet provider to maintain access to and functionality of study related software. These include a web-based randomization tool, a secure electronic database, online file share tools, and ECG device mobile apps. While the use of mobile phone technology has grown (29), the use of smartphones in Nigeria and across Africa is much lower largely due to cost (29). Low digital literacy levels in the general population (30) also limits patient's uptake of digital technology solutions. However, studies have shown that the general attitude towards health technology solutions is largely positive (31, 32). All study sites reported that the vast majority of participants and hospital staff were interested in and willing to use health AI technology. At LUTH, all eligible patients screened agreed to participate in the study representing a 100% enrollment rate at that site. This was largely attributed to the COVID pandemic which led to a shift towards virtual visits and the use of technology-based options to provide care.

#### Proposed solution to digital infrastructure, Literacy, and Internet connectivity

The establishment of public-private partnerships between the government and mobile network operators in Nigeria can enhance digital inclusion by expanding access to affordable broadband internet services. Ongoing efforts by the federal government include the creation of FMCIDE's national broadband plan which aims to initiate a national fiber optic network roll-out starting with specific regions in 2024 (11). Efforts to promote digital literacy through targeted education and skill building activities for healthcare professionals will be essential in ensuring successful implementation of AI and digital health technologies. Creating awareness of digital health technologies and educational programs for the general public can improve digital literacy levels and foster the adoption of AI tools for healthcare.

*F. Supply chain challenges:*

Medical device and equipment supply chain management also proved to be a huge challenge. Local country medical device teams were unable to assist with repairs or provide device replacements during the study due to a non-existent relationship with the U.S. based office. Attempts at acquiring 12-lead ECG machines with the required functionality (able to export raw digital files) in Nigeria through the local country office and other medical devices vendors were unsuccessful due to insufficient inventory.

#### Proposed solution to supply chain challenges

Market diversification can ensure that medical equipment and digital health devices are available from multiple sources. A competitive market can also lower equipment costs and limit price gouging. Interim strategies may include utilizing devices that are locally available in the country. However, this approach necessitates thorough validation of existing AI algorithms based on data collected from these devices before implementation.

### 2.2 Individual/patient factors

*A. Poverty/financial challenges:*

The poverty rate in Nigeria was estimated at approximately 40% in 2023, making it one of the world's poorest populations following India (27). It is projected that these rates will increase through 2025, given the recently enacted reforms directed at re-establishing macroeconomic conditions and facilitating economic recovery (27). Healthcare financing primarily relies on out-of-pocket payments, with few individuals having access to health insurance through government funded options, private insurance, or health maintenance organizations (HMOs).

#### Proposed solution to poverty/financial challenges

The importance of adequate healthcare financing in ensuring a viable health system cannot be overemphasized and government investments will be critical. Creating awareness of available government funded healthcare coverage plans and implementing measures to ease registration requirements for residents can substantially improve access to care. Simplifying existing processes by allowing automated registration for all interested parties seen at any clinical care site (including pharmacies, community clinics, and hospitals) can improve health insurance uptake. However, pending national health system reforms there are some opportunities to leverage existing structures for healthcare delivery in a cost-effective manner. Several private organizations provide various forms of healthcare access solutions through telehealth and remote monitoring options. These could be leveraged to provide home and community-based AI-guided screening to pregnant and post-partum women through community health workers, nurses or midwives, and potentially doulas, or traditional birth attendants who provide physical and emotional support to women in the perinatal period.

*B. Cultural norms, religious beliefs, and gender roles:*

Many women from the Northern sites (AKTH and RSSH) wear an hijab (head covering) or Abaya (covering for the entire body except the face, hands, and feet) as symbols of their faith and modesty. The garments can restrict the ability to perform physical examination and certain tests such as ECGs and echocardiograms. Tests requiring clothing removal had to be conducted by women or supervised by female chaperones. In sites without female staff, it was necessary to engage female nurses or hire female research assistants to ensure compliance with these requirements and to train them in obtaining standard and portable ECG recordings. For echocardiograms, sites without a female cardiologist or sonographer had female staff present as chaperones. Regarding mortality ascertainment, due to the absence of national death registries, data were obtained by reviewing health records and contacting relatives if the study participants were unreachable. However, cultural and/or societal norms pertaining to informing individuals who are not closely related to the deceased may affect how death status is reported. For example, one woman had been reported to the study team as “doing well but was out of town” during multiple follow-up calls. Finally, during a phone conversation with the participant's husband at the study closeout, it was revealed that the patient had passed away five months prior. The husband shared that relatives were reluctant to inform individuals outside the family about her death. Regarding a new diagnosis of cardiomyopathy, one of the themes that emerged among some women include spiritual and traditional beliefs about miraculous healing leading them to believe that they do not need medications. Additionally, there is a perception that disease states diagnosed in asymptomatic (apparently healthy) women are often inaccurate and do not require treatment. Particularly in the northern sites, traditional gender roles are prevalent with women being less likely to work outside the home and were reliant on their husbands for financial support. As such, healthcare decisions are often deferred to men (heads of household) which contributes to women experiencing delays in seeking care even when they wish to do so.

#### Proposed solution to cultural norms, religious beliefs, and gender roles

Health facilities should employ female staff across all care units to ensure culturally responsive and appropriate care, which includes understanding and respecting diverse cultural practices as it relates to health. Additionally, public health interventions aimed at increasing awareness of pregnancy-related cardiomyopathy and the significance of early treatment will be essential. This can involve the use of multiple media avenues, including television and radio awareness campaigns, community health awareness events, collaborations with religious institutions, and social media. Creating support groups led by trained peers and community leaders after diagnosis can provide psychosocial support to women diagnosed with peripartum cardiomyopathy, offering them a safe space to discuss concerns with others who share similar cultural values. To address gender related barriers to seeking care, we suggest public awareness initiatives encouraging education for girls, laws and regulations to discourage child marriage, offering remote vocational opportunities for women, and utilizing impactful visual campaigns to challenge existing gender roles.

### 2.3 Technical factors

*A. Hardware device related challenges:*

**Figure 2:**
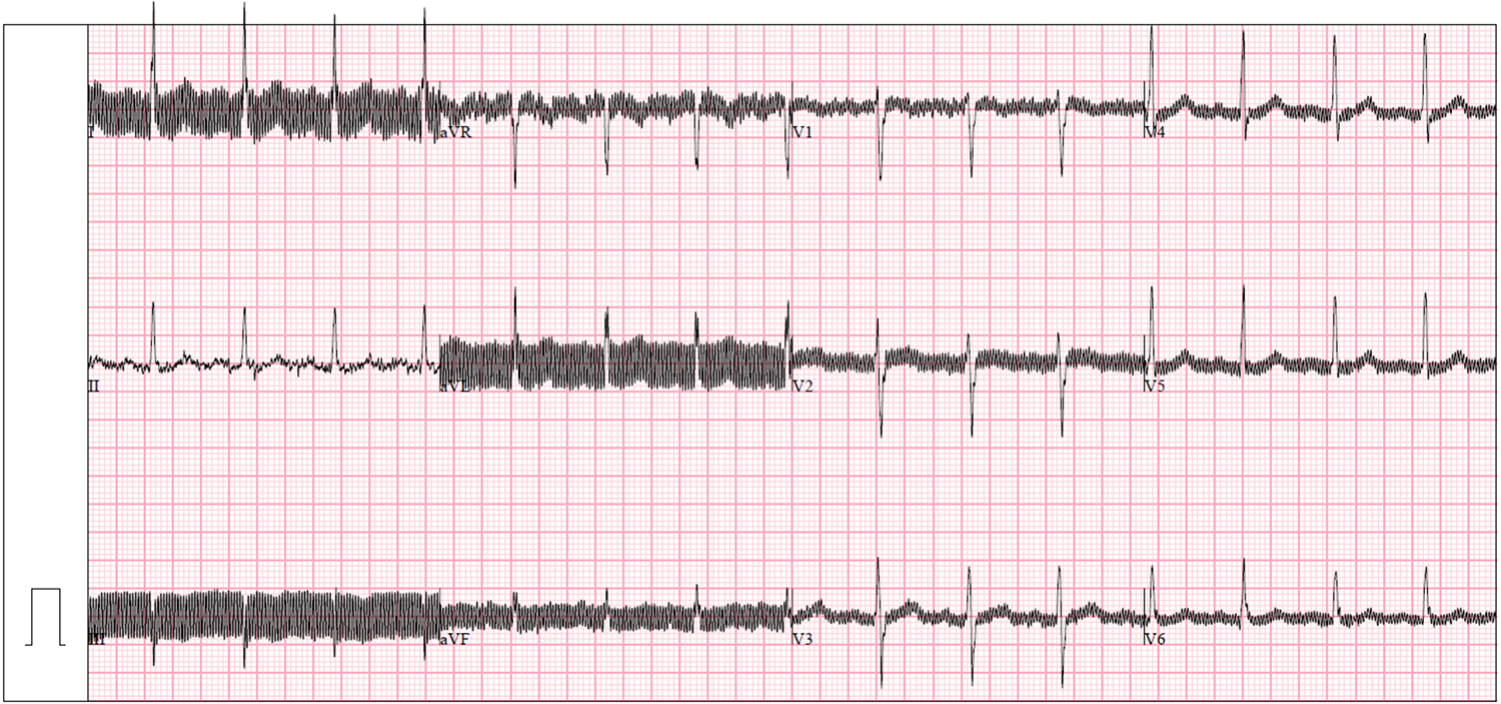
Sample ECG with electromagnetic interference-like artifact.

The devices used in the study were purchased in the United States. They operate on standard electrical voltages ranging from 110 to 120 V (60 Hz). However, in Nigeria, the standard electrical voltage range is 220–240 V (50 Hz). Although the device specifications state that the allowed input voltage ranges from 100 to 240 V, two of the 12-lead ECG machines malfunctioned during the study, suspected to be related to fluctuations in the power grid. In addition, we observed artifacts resembling electromagnetic interference on the ECGs (Figure 2) early during the study, particularly when the device was plugged in or in close proximity to an echocardiogram machine in the same room.

#### Proposed solution to hardware device related challenges

Regarding ECG artifacts, initial troubleshooting attempts were unsuccessful and the solutions that worked were to operate the 12-lead ECGs with battery power (avoiding plugging into an outlet during ECG recording) and, when possible, recording the ECGs in a different room from the echocardiogram machine. It is important to consider devices that have long-lasting battery power so that these can be used during power outages. In the long term, government-led strategic partnerships with industry to ensure that high-quality diagnostic medical devices are locally available and exploring avenues to increase local production and device assembly will lead to significant economic benefits and improved health outcomes by reducing the need for imports and lowering costs. The use of devices acquired in-country also ensures that appropriate voltage requirements are in-built, which reduces the likelihood of power grid-related damage.

*B. Software related challenges:*

We discovered prior to study launch that the mobile apps required for the digital health devices (digital stethoscope and portable ECG) were not available on the Apple App Store or Google Play Store for the Nigerian region. Without the accompanying mobile app, these devices are essentially non-functional. To ensure the successful conduct of the study, we purchased tablets in the U.S. and pre-downloaded the necessary mobile apps from the U.S. Apple App Store. Additionally, the tablets were programmed with mobile device management technology, enabling remote management and troubleshooting. Prior to the completion of the study, the specific mobile apps used were made available for download in Nigeria which will facilitate the use of these digital health technologies moving forward. The decisions made by technology companies regarding the availability of health-related mobile apps in different regions of the world can have a significant health impact, especially in economically disadvantaged areas. One factor contributing to this decision is a presumed low return on investments and this can be described as a form of global digital redlining. Digital redlining refers to practices that result in unequal access to digital resources and opportunities across different regions.

#### Proposed solution to software related challenges

It is imperative that governments and international organizations such as the United Nations (UN) and World Health Organization (WHO) support healthcare technology expansion to low- and middle-income countries (LMIC). Efforts by WHO to help countries negotiate lower prescription drug prices, hold pharmaceutical companies publicly accountable, and ensure generic formulations are made available (33) can serve as a model for ensuring the availability and affordability of specific health-related software and digital tools. Other examples include public-private partnerships, such as the meningitis vaccine project. This project led to the development of a low-cost meningitis vaccine for use in Africa, facilitated by African scientists working with an international consortium of academics (33). The proposed strategies would help ensure that the world's population, particularly those in LMIC, can access digital health innovations equitably, which are often limited to wealthier Western nations. In the interim, alternative solutions to accessing critical health-related mobile apps and software not available in a specific country include the use of a virtual private network (VPN), if there are no country-specific legal restrictions on its use.

## 3 Conclusion

We provide a detailed summary of the contextual barriers and challenges to healthcare AI implementation in a low-resource environment. Additionally, we suggest solutions and strategies to guide implementation based on lessons learned from the recently completed SPEC-AI Nigeria clinical trial—a randomized controlled clinical trial of an AI intervention (13). As the field of healthcare AI gradually expands across the globe, understanding the challenges faced by developing countries can inform implementation strategies and help develop effective approaches to ensure successful integration within health systems.

## Data Availability

The original contributions presented in the study are included in the article/Supplementary Material, further inquiries can be directed to the corresponding author.
